# Stroke and migraine is there a possible comorbidity?

**DOI:** 10.1186/s13052-016-0253-8

**Published:** 2016-04-26

**Authors:** Alberto Spalice, Francesca Del Balzo, Laura Papetti, Anna Maria Zicari, Enrico Properzi, Francesca Occasi, Francesco Nicita, Marzia Duse

**Affiliations:** Department of Pediatrics Child Neurology Division, Sapienza University Rome, Viale Regina Elena 324, 00161 Roma, Italy

**Keywords:** Migraine, Stroke

## Abstract

The association between migraine and stroke is still a dilemma for neurologists. Migraine is associated with an increased stroke risk and it is considered an independent risk factor for ischaemic stroke in a particular subgroup of patients. The pathogenesis is still unknown even if several studies report some common biochemical mechanisms between these two diseases. A classification of migraine-related stroke that encompasses the full spectrum of the possible relationship between migraine and stroke includes three main entities: coexisting stroke and migraine, stroke with clinical features of migraine, and migraine-induced stroke. The concept of migraine-induced stroke is well represented by migrainous infarction and it is described in the revised classification of the International Headache Society (IHS), representing the strongest demonstration of the relationship between ischaemic stroke and migraine. A very interesting common condition in stroke and migraine is patent foramen ovale (PFO) which could play a pathogenetic role in both disorders. The neuroradiological evidence of subclinical lesions most typical in the white matter and in the posterior artery territories in patients with migraine, opens a new field of research. In conclusion the association between migraine and stroke remains an open question. Solving the above mentioned issues is fundamental to understand the epidemiologic, pathogenetic and clinical aspects of migraine-related stroke.

## Background

Migraine is a common, chronic, multifactorial neurovascular disease, characterized by severe attacks of headache and autonomic nervous system dysfunction. Migraine is common among children and adolescents. Its prevalence has a documented range from 0.5 to 13.6 % and increases with age. Sex distribution is almost equal between ages of 7 and 11. During puberty, the balance shifts to a 3:1 ratio between females and males, which extends into adulthood. The two most common types of migraine in children are migraine without aura (MO) and migraine with aura (MA). Childhood periodic syndromes (cyclical vomiting, abdominal migraine and benign paroxysmal vertigo of childhood) frequently precede migraine and occur exclusively in the pediatric population [1–4].

Migraine without aura, previously known as “common migraine”, is the most frequent type of migraine, accounting for 60–80 % of all migrainous headaches. Children experiencing migraine without aura have often prodromal symptoms such as behavioral changes (e.g. irritability or decrease in energy) and appearance abnormalities (e.g. pallor, pigmented macules in the infraorbital region or “dark rings under the eyes”). Migraine with aura is characterized by occurrence of one or more fully reversible auras before the onset of headache. Children may complain of visual disturbances (visual auras) and hallucinations. Somatosensory aura, though uncommon, consists of perioral paresthesias and/or numbness and tingling of hands or feet (or both) [1–5].

Although migraine attacks may be acutely disabling, they do not result in long-term brain consequences.

Against this assumption, new data have emerged emphasizing migraine high prevalence among young individuals with stroke, dysfunction of cerebral arteries during migraine attacks and incidence of silent infarct-like brain lesions in migraineurs. It suggests thus that it might exists a comorbidity between migraine and cerebral ischemia [6–8].

## Classification of migraine related stroke

One of the most relevant drawbacks in unrevealing the complex relation between migraine and cerebral ischemia is the lack of consistency while defining migraine-related stroke.

Attempting a categorization, four major issues should be considered:

- cerebral ischemia can occur during an attack of MA, causing true migraine induced infarction;
- migraine and stroke share a common underlying disorder increasing the risk for both diseases;
- migraine might cause stroke only as far as other risk stroke factors are present interacting with the migraine induced pathogenesis;
- stroke can mimic migraine [1–7].

The Table 1 presents an extended classification of stroke associated with migraine or migraine-related stroke.

**Table 1:** Possible comorbidities between migraine and stroke

Type	Definition
Migraine as a risk factor for stroke	A clearly clinically defined stroke syndrome must occur remotely in time from a typical attack of migraine
Migraine caused by stroke (symptomatic migraine)	An acute vascular event in the central nervous system (ischemic or hemorrhagic stroke or TIA) produces episodes of headache with the characteristics of migraine with or without aura; to be coded as ICHD-II 6.1
Migraine as a caused of stroke (migrainous infarction)	A documented infarct in a relevant area during the course of an attack of migraine with aura, in a patient with a history of migraine with aura, with symptoms that are those of the aura and in the absence of other possible causes at an extensive workup; to be coded as ICHD-II 1.54
Migraine and stroke sharing a common cause	A syndrome (usually of genetic origin) in which both migraine and stroke are major clinical features (e.g. CADASIL [ICHID-II 6.7.1] or [ICHD-II] 6.7.2)
Migraine associated with subclinical stroke	Evidence at brain neuroimaging of small areas compatible with brain ischemia in patients without a history of any clinical symptom indicating a stroke syndrome
Migraine mimicking stroke (and vice versa: stroke mimicking migraine)	Symptoms of migraine attacks (particularly aura without headache) and of stroke (particularly TIAs) may overlap causing problems in the differential diagnosis

*TIA* transient ischemic attacks, *ICHD-II* International Classification of Headache Disorders, Second Edition, *CADASIL* Celebral Autosomal Dominant Arteriopathy with subcortical Infarcts and Leucoencephalopathy; *MELAS* Mitochondrial Encephalopathy, Lactic Acidosis and Stroke like episodes

### Migraine-induced stroke: the migrainous infarction

According to the International Headache Society (IHS) migraine classification, ‘migrainous infarction’ is defined as a stroke occurring during a typical attack of MA [1].

HIS criteria for migrainous infarction are:

- One or more migrainous aura symptoms not fully reversible within 7 days and/or associated with neuroimaging confirmation of ischemic infarction.
- Patient has previously fulfilled criteria for migraine with neurologic aura.
- The present attack is typical of previous ones, but neurologic deficits are not completely reversible within 7 days and/or neuroimaging demonstrates ischemic infarction in the relevant area.
- Other causes of infarction ruled out by appropriate investigations.

According to large series, the incidence of migrainous infarction varies between 0.5 and 1.5 % of all ischemic strokes and 10 to 14 % of ischemic strokes in young patients. Incidence of migraine-related infarction (per 100,000 persons per year) was estimated at 1.44 (95 % CI, 0 to 3.07) from the Oxford shire Community Stroke Project prospective registry [7–11].

### Migraine and stroke share a common cause

Several vascular disorders can cause stroke and are associated with a high risk of migraine, mostly MA. Arteriovenous malformations (MAV) are the classical cause of symptomatic migraine. A causal relationship is supported by the side of the aura being contralateral and the headache being ipsilateral to the MAV. Other MA potentially associated conditions include leptomeningeal angiomatosis (Sturge Weber syndrome) and hereditary haemorrhagic teleangectasia.

Ischemic stroke and migraine are also major clinical features of some specific syndromes: cerebral autosomal-dominant arteriopathy with subcortical infarcts and leukoencephalopathy (CADASIL), mitochondrial myopathy, encephalopathy, lactic acidosis, and stroke-like episodes (MELAS), cerebroretinal vasculopathy, and hereditary endotheliopathy with retinopathy, nephropathy, and stroke (HERNS). Coexistence of ischemic stroke and migraine in a syndrome with a peculiar phenotype, a confirmed inheritance and chronic alterations of the wall of cerebral small-vessel arteries, highlights a common pathogenic mechanism between these two conditions [1–6].

Ischaemic strokes and MA might also occur in many general vascular disorders: cardiac disorders such as patent foramen ovale and mitral valve prolapse, and blood disorders such as essential thrombocythaemia, thrombocytopenia, leukaemia, and systemic lupus erythematosus. Co-morbidity can explain some cases others by ischaemia-induced MA attacks and biochemical factors such as serotonin changes in platelet disorders or some immunological changes, especially antiphospholipid syndrome and systemic lupus erythematosus [1–12].

### Stroke miming migraine

Transient ischemic attacks (TIA) and migraine auras are both characterized by temporary focal neurological deficits; starting from patient’s description, differential diagnosis become clear when symptoms are typical. In migraine auras, positive symptoms such as scintillations progress gradually over several minutes and last about 30 min, after which commonly occurs a severe headache. In transient ischemic attacks, there is a focal deficit of sudden onset typically lasting less than 15 min, without ensuing headache. MA typically starts in childhood, whereas transient ischemic attacks tend to occur in adulthood. Nevertheless, migraine can present with TIA-like symptoms (eg, haemianopia without headache) for the first time after 40 years of age. Moreover, transient ischemic attacks, particularly basilar transient ischemic attacks, can sometimes be associated with headache. Mode of onset becomes a crucial distinctive clinical feature [1–5]

Spontaneous cervical artery dissection (sCAD) is a good example of a migraine mimic, especially because patients with migraine are at increased risk of dissection. These symptomatic migraine attacks might be more common than migraine-induced ischemic insults. Cerebral infarction can thus present with migraine attacks at onset, which should not be confused with migrainous infarction. A stronger association was observed among younger female, whose ischemic event was located in the vertebrobasilar territory, with a personal history of migraine [11–14].

### Coexisting ischemic stroke and migraine

Despite the apparent increased risk of stroke in migraineurs, the high frequency of migraine in young adults underlines its possible coexistence with ischemic stroke without contributing to its development.

Being the etiology of these strokes probably multifactorial, identification and management of other established risk factors should be pursued with particular vigilance in all migraine sufferers [11–13].

## Pathogenesis of migraine induced stroke

The mechanism of IS increased risk in migraine is unknown and numerous hypotheses have been raised.

### Hemodynamic factors

Migraine is considered a neurovascular disorder with arterial constriction and posterior circulation decreased blood flow as consequences of spreading wave of neuronal depression in the cerebral cortex. In this regard, Cortical Spreading Depression (CSD) may induce cerebral blood flow short-lived increases and tissue hyperoxia, followed by deeper oligoemia and consequent increased intraparenchymal vascular resistance. Thus, low flow in major intracerebral vessels may be due to increased downstream resistance and not to major intracranial arterial vasospasm. A low cerebral blood flow and neuronally mediated vasodilatation could then cause sluggish flow in large intracerebral vessels during migraine auras. When combined with coagulopathy predisposing factors, as dehydration hyperviscosity or intravascular thrombosis, migraine-induced cerebral infarction may occur, even if rarely [11–15].

### Inflammatory factors

CSD is accompanied by vasodilatation of extrapaenchymal vesels and release of neuronal inflammatory mediators. Releasing of vasoactive peptides and nitic oxide, activation of cytokines, and adhesion molecules upregulation also predispose to intravascular thrombosis. This could explain why migraine-induced stroke usually respects intracranial arterial territories whereas aura involves more widespread brain regions. Moreover, frequent aura due to CSD, could induce cytotoxic cell damage and gliosis based on glutamate release or excessive intracellular calcium accumulation. Thus, a persistent neurologic deficit could be due to selective neuronal necrosis.

Vasospasm was previously thought to be implied in migraine aura, resulting from releasing of vasoconstrictive molecules as endothelin and serotonin. In rare documented cases it was involved in migrainous infarction [15–18].

### Coagulation factors

Experimental data point toward activation of the thrombotic cascade during a migraine attack. Indeed, platelets and mast cells showed to release platelet activating factor (PAF), a potent inducer of platelet activation and aggregation. PAF is also involved in the release of von Willebrand factor, and indirectly in the activation of the platelet IIb/IIIa receptor, crucial for binding fibrinogen thus leading to primary hemostasis.

Increased plasma levels of these molecules have been observed during the course of migraine attacks compared with those of interictal phases [11–15]

However, these mechanisms apply only for the so called migrainous strokes, which, as defined by the IHS criteria, are a rare event. This low incidence cannot explain then the increased risk of stroke in migraine [11–17].

### Migraine and patent foramen ovale

Patent foramen ovale (PFO), an interatrial communication remnant of the fetal circulation, is due to a failure in the fusion between septum primum and septum secundum. The prevalence of PFO in healthy adult population is around 25 %. Several case–control studies observed that patent foramen ovale (PFO) is significantly more common in patients who suffered MA than those without migraine. Right-to-left cardiac shunt at rest through a PFO is more common in migraineurs with aura than in non-migraineurs control patients with PFO and patients with migraine tend to have greater right-to-left shunts as compared to the non-migrainous population. This suggests that interatrial communication may play a role in the pathogenesis of migraine. The mechanisms underlying this possible association was postulated but never demonstrated. Paradoxical embolism is suggested to be the causal link between migraine and PFO, but data available are insufficient to substantiate the hypothesis that migraine frequency (and, indirectly, ischemic stroke risk) is reduced by PFO closure. A variable proportion of patients who underwent PFO closure for non-migraine indications however reported cessation or improvement of their migraine attacks after the procedure. On the basis of these findings, the possibility of a PFO–migraine– ischemic stroke triangular association remains a matter of speculation [15–18].

### Vascular factors

In the last years, some observations have suggested migraine as a predisposing condition for sCAD, one of the most common causes of stroke in young patients. The mechanism by which migraine may affect the risk of sCAD is unknown. A generalized vascular disorder is hypothesized to be a predisposing condition for both diseases. Recent observations in migraineurs of increased activity of serum elastase, a metallopeptidase that degrades specific elastin-type amino acid sequences, suggest a possible extracellular matrix degradation facilitating sCAD occurrence. In line with previous observations of altered common carotid artery distensibility in patients with sCAD, it was recently reported that an endothelium-dependent vasodilatation assessed in the brachial artery is significantly impaired in these subjects. Similar vascular changes were observed in migraine patients during interictal periods and replicated in a recent cross-sectional study in migraineurs of recent onset, excluding the possibility of a bias due to longstanding history of migraine and repeated exposure to vasoconstrictor drugs. Finally, the analysis of small families has shown that the structural abnormalities related to sCAD might be familial and follow an autosomal-dominant pattern of inheritance. This suggests that genetically determined alterations of the extracellular matrix may play a crucial pathogenic role and that candidate genes involved in endothelial and vessel wall functions regulation might increase susceptibility to both conditions [18, 19].

### Endothelial factors

Inconsistent results have been found for the various biologic or clinical markers of thrombotic risk studied so far, such as platelet activation, factor V Leiden mutation, von Willebrand factor, prothrombin factor 1.2, platelet leukocyte aggregation, antiphospholipid antibodies, and livedo reticularis. In contrast, there is mounting evidence that migraine may be a risk factor for endothelial dysfunction, a possible link to ischemic stroke and heart disease.

Endothelial dysfunction is characterized by reduction in bioavailability of vasodilators (such as nitric oxide), increase in endothelial derived contracting factors, and consequent impairment of vessels reactivity, including the microvascular system. It represents the first step in the development of atherothrombosis finally leading to vascular events. It also comprises endothelial activation, characterized by a procoagulant, proinflammatory and proliferative state, which in turn predisposes to ischemia. Endothelial dysfunction is mediated by increased oxidative stress, an important promotor of the inflammatory process, that might have a role in the pathogenesis of migraine. Clinical investigation of markers of oxidative stress in a migraine population during, after, and between migraine attacks yielded support for the association. In fact, compared with migraine-free controls, oxidative stress markers were found higher in migraineurs, even during the interictal period, yielding support to the association [12–14].

Reduced endothelial repair capacity has emerged as another possible connection between migraine and vascular disease. Levels of endothelial progenitor cells, measured using flow cytometry, were found lower in migraineurs – particularly in those with aura – if compared to healthy controls and to patients with tension-type headache. Patients with migraine presented also increased markers of senescence and decreased migratory capacity of endothelial progenitor cells. Endothelial progenitor cells derive from bone marrow, circulate in peripheral blood, are capable of proliferation and differentiation into endothelial cells and play a role in neoangiogenesis after ischemia. Although it is not known if the reduction of endothelial progenitor cells represents a primary alteration in migraine or the consequence of migraine attacks itself, it is possible that their alteration mediates an increased vascular risk [12–19].

### Medications effects

Raised risk due to migraine treatments , particularly vasoconstrictors, is supported by an increase in white matter abnormalities and in mortality found in patients taking ergotamine, though recent studies found no increase in severe vascular events treated with triptans. Furthermore, drugs widely used in migraine, such as non-steroidal inflammatory drugs, decrease the risk of cerebral ischemic events [20].

## Genetic influence on migraine-stroke relation

Over the past years, evidence from twins and family history studies, although not entirely consistent, has supported the notion that genetic predisposition plays a major role in the occurrence of both migraine and ischemic stroke.

### Monogenic forms of migraine

Even if many chromosomal regions were reported as possibly involved in migraine occurrence, mutations in three genes for familial hemiplegic migraine (FHM) represent the only established monogenic cause of migraine so far. Familial hemiplegic migraine is a subtype of MA characterized by an autosomal-dominant pattern of inheritance and at least some degree of weakness (hemiparesis) during the aura. In spite of these clinical markers, a broad variability rules: age at onset, frequency, duration and features of attacks may differ from one patient to another, even among affected members from a family carrying the same mutation in the same gene. Less frequent features such as cerebellar ataxia, occurring in some families, minor head trauma acting as triggering factor and severe attacks with impairment of consciousness have also been reported. Moreover, even headache has been reported as the sole clinical manifestation [2, 21]. Furthermore, the majority of FHM patients experience attacks of typical MA and MO. It seems then reasonable to assume that migraine is composed by a spectrum of pathologic identities, starting from the common forms of migraine up to FHM. The latter is thus a valid model to study genetic factors of migraine in general as well as the relation between migraine and ischemic stroke. To date, three different genes responsible for different subtypes of FHM have been identified:

- FHM1 is caused by mutations in the CACNA1A gene, located on chromosome 19p13, encoding the pore-forming a1A subunit of Cav2.1 (P/Q type) voltage-gated neuronal calcium channels;
- FHM2 is caused by mutations in the ATP1A2 gene, located on chromosome 1q23, encoding the a2 subunit of sodium–potassium pump ATPases;
- FHM3 is caused by mutations in the SCN1A gene, located on chromosome 2q24 encoding the a1 subunit of the neuronal voltage-gated sodium channel Nav1.1,crucial in action potentials generation and propagation.

Overall, the common consequence of FHM1, FHM2, and FHM3 mutations seems to be to increased levels of glutamate and potassium in the synaptic cleft causing an increased propensity to CSD. Whether this might also increase the propensity to cerebral ischemia is unknown. Similarly, the contribution of FHM genes in common forms of migraine (MO and MA) remains unclear. A recent study showed no linkage to the CACNA1A and ATP1A2 genes in families with apparently autosomal – dominant mode of inheritance of MA, whereas a case – control study investigating the role of the ATP1A2 gene in MA found no evidence of association [11, 12].

### Polygenic forms of migraine

New powerful technologies of gene analysis along with the possibility to use informatics resources providing genome-wide sequence and variant data has fostered an effective and challenging approach to complex diseases. On the basis of the results of such analysis, several specific genetic variants have been implicated in migraine susceptibility and it can be gathered from three main streams.

- The first group includes genes involved in neurotransmitter-related pathway, such as genes encoding for dopamine D2 receptor (DRD2), human serotonin transporter (HSERT), catechol-O-methyltransferase (COMT), and dopamine b-hydroxylase (DBH).
- The second group includes genes involved in vascular function, such as 5,10-methylenetetrahydrofolate reductase (MTHFR), angiotensin I-converting enzyme (ACE), and endothelin type A (ETA) receptor.
- The third group includes genes involved in hormonal function, such as estrogen receptor 1 (ESR1), progesterone receptor (PGR) and androgen receptor (AR).

Several genes candidate for migraine are also good candidate for cerebral ischemia. Among them, in spite of inconsistent results of some studies hypothesizing a link between this marker and migraine, C677T polymorphism of MTHFR gene looks particularly promising, due to its probable independent effect on ischemic stroke risk. This enzyme catalyzes the reduction of 5,10-MTHF to 5-MTHF, the circulatory form of folate and carbon donor for re-methylation of homocysteine (Hcy), a reactive thiol amino acid, to methionine. It was found that with low dietary folate, patients with migraine and MTHFR C677T variant homozygosis have a higher risk for elevated Hcy (hyperhomocysteinemia or HHcy) levels. HHcy is defined as Hcy level above 5–15_mol/L. Elevated Hcy levels were reported in patients with MA. Animal studies suggest that Hcy may increase migraine susceptibility by heightening cerebral artery sensitivity. Hcy-related endothelial dysfunction seems involved in migraine beginning and maintenance of. Endothelial dysfunction may result from direct damaging effect of Hcy on endothelium and altered oxidative status. Hcy is indeed a risk factor for endothelial cell injury, atherosclerotic vascular diseases, independent of the long-recognized identified common risks. Autooxidation of Hcy promotes the production of hydroxyl radicals, thiolactone and known lipid peroxidation initiators with creation of a prothrombotic environment. It is possible that HHcy may result in temporary cerebral thrombosis and/or altered blood flow, allowing less oxygen into the brain and manifesting the common symptoms to MA and ischemic stroke [12, 13].

## Neuroimaging

Abnormalities of uncertain clinical significance are frequent findings on brain MRI scans in patients with migraine. The most common abnormality is white matter lesions (WMLs), typically multiple, small, punctate hyperintensities, occurring in the deep or periventricular white matter and often seen on T2-weighted or Fluid-Attenuated Inversion Recovery (FLAIR) images. In a small minority of cases, number, distribution, and location of WMLs may lead to the diagnosis of an underlying disease of which migraine may be but one symptomatic manifestation. Some authors hypothesized that white-matter abnormalities are due to ischaemic insults which leed to the suggestion that migraine could be a progressive brain disorder [12].

## Conclusion

The bidirectional relation between migraine and ischaemic stroke, though poorly understood, has important practical implications. To date, available data support the following recommendations:

- Emphasis on identification and treatment of modifiable vascular risk factors, such as smoking, hypertension, diabetes, and hypercholesterolemia, is warranted in migraineurs, especially those with MA.
- Because of the potential synergistic effect of several migraine-specific drugs with vasoconstrictive action, including triptans, and traditional predisposing conditions in increasing the risk of ischemic stroke, subjects with major cardiovascular risk factors should be encouraged to adopt migraine prophylactic strategies. This approach should be also recommended to subjects with a personal history of prior ischemic disease (cerebral and/or myocardial).
- There is no direct evidence that PFO closure is effective for MA prophylaxis and, indirectly, for primary prevention of stroke, and, this procedure cannot thus be recommended for MA prophylaxis.
- Patients with migrainous stroke should undergo the same diagnostic workup and receive the same pharmacological treatment of any ischemic stroke in the young, both during the acute phase and follow-up.
- Preventing disease progression in migraine has already been added to traditional goals of relieving pain and restoring patients’ ability to function. If migraineurs brain lesions of have a significant clinical correlation, preventing brain lesions accumulation may become an additional goal of treatment. The association of stroke with frequency of migraine attacks suggests that migraine prophylaxis, especially MA, may actually reduce migraine-related stroke risk. It opens then issues of whether prophylactic drugs decreasing such a risk (i.e., antihypertensives) might be the best choice in these cases [17–20].
